# Thermodynamics of climate change between cloud cover, atmospheric temperature and humidity

**DOI:** 10.1038/s41598-021-00555-5

**Published:** 2021-10-28

**Authors:** Víctor Mendoza, Marni Pazos, René Garduño, Blanca Mendoza

**Affiliations:** 1 Instituto de Ciencias de la Atmósfera y Cambio Climático, Universidad Nacional Autónoma de México, C.P. 04510 Mexico City, Mexico; 2 Escuela Nacional de Ciencias de la Tierra, Universidad Nacional Autónoma de México, C.P. 04510 Mexico City, Mexico; 3 Instituto de Geofísica, Universidad Nacional Autónoma de México, C.P. 04510 Mexico City, Mexico

**Keywords:** Climate sciences, Atmospheric science, Atmospheric dynamics

## Abstract

On a global and annual average, we find a parameterization in which the cloud cover increase is proportional to the mid tropospheric temperature increase, with a negative proportionality factor. If the relative humidity is conserved throughout the troposphere, a 1 °C heating (cooling) of the mid troposphere, decreases (increases) the cloud cover by 1.5 percentage points (pp). But if the relative humidity is not conserved, then the cloud cover decreases (increases) by 7.6 pp. If the shortwave reflection effect of the cloud cover is dominant on a global scale, this parameterization leads to a predominant positive feedback: if the temperature increases like in the current climate change, the cloud cover decreases and more solar radiation reaches the surface increasing the temperature even more. The contribution of the present work consists in finding that the negative sign of the proportionality factor is due to the Clausius–Clapeyron equation; that is, to the magnitude of the derivative of the saturation vapor pressure at the typical standard surface temperature of 288 K. The negative sign of the factor is independent on the conservation or non-conservation of relative humidity in the troposphere under climate change.

## Introduction

Atmospheric climate models (CMs), along with observations, are some of the main tools for weather prediction and climate research. Laws of conservation of energy, momentum, and mass describe the thermodynamics and dynamics of the different processes simulated by the CMs^1^. In this way, physical processes are ideally represented by these conservation laws, however, when the processes cannot be represented on certain scales of space and time, semi-empirical parameterizations are necessary to have as many equations as unknowns.

Early works addressed the importance of a proper cloud parameterization^2,3^. More recently, it has been mentioned that one of the shortcomings in CMs are the representation of clouds, precipitation, and circulation^4^. Depending on the CM, and even in the simplest model configuration, the patterns of clouds and precipitation response to warming might have substantial variations^5^.

The cloud role in climate consists in modifying the radiation budget regionally and at planetary scales, as clouds impact in both shortwave and longwave radiation^2^. Besides characterizing the weather, clouds also have and important effect on the dynamics and structure of weather systems, such as precipitation distribution and tropical and extratropical circulation^4^.

The importance of cloud cover parameterization can be found in several works^6–8^, addressing that the aerosols produced by desert dust acting as cloud condensation nuclei indirectly influence the cloud cover impacting the Earth’s energy budget by scattering and absorbing solar radiation, absorbing, and emitting thermal outgoing radiation. The cloud-aerosol interactions and total cloud cover calculation are also a large source of uncertainty, which may cause large bias in radiation simulation. Models that simulate a higher-than-average warming also simulate a larger than average shortwave net increase, along with a higher-than-average reduction of the cloud screening (albedo) effect^9^. To numerically simulate the response to warming, should also be considered the phenomenon of the warming-trend slowdown, a hiatus in global warming probably due to natural variability^10^.

Since early 1980s it has been recognized, as a good approximation, that the tropospheric relative humidity remains constant in a climate change, mainly over oceanic regions near the surface^11–14^.

The cloud formation processes in modern climate models (such as CMIP5), implies the development of several parameterizations that interact with each other. These models include the parameterization of turbulence, convective dynamical systems, cloud microphysics and radiative transfer processes, as well as evaporation, condensation and precipitation, among other parameterizations on which cloud formation depends; however, some of these complicated processes are unrealistic and therefore the response of clouds to climate change remains uncertain^14–16^.

In this order of ideas, applying the principles of thermodynamics, we present the formulation of two simple semi-empirical parameterizations that determine the decrease of the total cloud cover (ε; which includes low cloud, middle cloud and height cloud) in terms of the tropospheric temperature increase (the terms decrease or increase denoted by ∆ should be understood as changes of thermodynamic state).

We consider that on a global and long-term scale, these parameterizations can reduce the uncertainty in the response of clouds to an increase in global temperature and therefore deserve further scientific research that is based mainly on thermodynamics. The first parameterization is based on the conservation of relative humidity mentioned above. The second takes into account the increase in precipitable water and the use of the Clausius–Clapeyron equation for two cases: when the relative humidity is conserved and not conserved, considering in both cases that the temperature is increased. The two parameterizations can be used in CMs based on the thermal energy balance; in fact, the first parameterization has been used in the Thermodynamic Climate Model (TCM).

## Method

The TCM is based on the equation of thermal energy conservation or First Law of the Thermodynamics applied to: the troposphere with an average thickness of ~ 11 km and with a variable cloud cover generated by the condensation of water vapor, the ocean mixed layer with ~ 60 m depth, and the continental surface layer of negligible thickness; for surface temperatures below 0 °C, an albedo associated with a surface layer of ice or snow is included in the model. The TCM has three main (internal) feedback mechanisms due to: the atmospheric water vapor, the cloudiness and the surface cryosphere; all of them have been included through semiempirical parameterizations^17^. Here, we only describe the cloudiness feedback.

## First parameterization

Previously and collaterally to the TCM, Adem^18^ parameterized the tropospheric relative humidity ${f}^{*}$ (the asterisk meaning that the variable depends on the altitude z) as a function of cloudiness and temperature, to compute the normal monthly precipitable water over the Northern Hemisphere (NH) and the annual global vertical profile of atmospheric humidity^19^. Both results agreed well with observations.

On the NH troposphere, Adem^18^ found that the vertical average ($${f}_{m})$$ of $${f}^{*}$$ is linearly correlated to $$\varepsilon$$, and that the deviation $$({f}^{*}-{f}_{m})$$ is a quadratic function of the vertical coordinate $$(z)$$, this quadratic function is also verified in Mendoza et al.^20^ with annual and hemispheric average $${f}^{*}$$ data at various levels, obtained from the NCEP/NCAR Reanalysis (http://www.esrl.noaa.gov/psd/data/timeseries/). The adjustment coefficients of $$({f}^{*}-{f}_{m})$$ depend on the latitude and the season (Table 1 in Adem^18^). Garduño and Adem^21^ and Adem and Garduño^17^ took the hemispheric and annual average of these coefficients, assumed the tropospheric lapse rate ($$\beta$$) constant, changed the variable *z* by the variable temperature (*T*′), and found a relationship of $${f}^{*}$$ quadratic on T*, which can be expressed by:

1 $${f}^{*}={f}_{m}+{\alpha }_{0}+{\alpha }_{1}{T}^{*}+{\alpha }_{2}{T}^{*2}$$

where ${\alpha }_{0}$, ${\alpha }_{1}$ and ${\alpha }_{2}$ are parameters that depend on $\beta$ and the air surface temperature ${T}_{a}$. According to Eq. (1), during winter and in tropical latitudes (25°N), in where a typical value of ${f}_{m}$ is 54.9%^22^, so that on the surface where the temperature is 288 K, ${f}^{*}=76.48\%$. In approximately 7 km, where the temperature is 242.5 K, ${f}^{*}$ reaches its minimum value of 45.82%, and then begins to increase due to the decrease in temperature, until reaching the value of 52.50% in 11 km where the temperature is 216.5 K.

Assuming that Eq. (1) represents a thermodynamic state of the atmosphere at a certain altitude, where the temperature is T*, then a differential or quasi-static thermodynamic process for ${f}^{*}$, can be expressed as:

1′ $$d{f}^{*}=d{f}_{m}+\left({\alpha }_{1}+2{\alpha }_{2}{T}^{*}\right)d{T}^{*}$$

Integrating Eq. (1′) from the state ${f}_{m}$ and ${T}^{*}$ to the state ${f}_{m}+\Delta {f}_{m}$ and ${T}^{*}+\Delta {T}^{*}$, considering an finite temperature increases $\Delta {T}^{*}$, such that $\mid\Delta {T}^{*}\mid/{T}^{*}\ll 1$ and applying the hypothesis of fixed relative humidity ($d{f}^{*}=0$), we obtain:

2 $$\Delta {f}_{m}=-\left({\alpha }_{1}+{2\alpha }_{2}{T}^{*}\right)\Delta {T}^{*}$$

Adem^18^ established a linear correlation between ${f}_{m}$ and $\varepsilon$ given by:

3 $${f}_{m}={B}_{2}\varepsilon + {B}_{1}$$

If ${f}_{m}$ and $\varepsilon$ are in percent, then the values of ${B}_{2}$ is constant and is equal to 0.5 and ${B}_{1}$ is a parameter depending on season; equal to 24.0 in autumn and summer, 21.0 in spring and 25.5 in winter. The values of ${f}_{m}$ and $\varepsilon$ used in Eq. (3) are obtained from London^22^, who comments that observations of clouds from surface points constantly underestimate the amount of medium and high clouds, so that to correct this error the assumption is made that the medium and/or high clouds exist in the same proportion, even when they are covered by low clouds. London^22^ mentions that this assumption usually increases the amount of medium clouds by about 2–3% and the amount of high clouds by about 5–8%. Except in winter, Eq. (3) is not valid in lower latitudes: below 20°N in spring and summer and below 10°N in fall; for winter it is not valid above 70°N. In Eq. (3), the correlation coefficients for spring, summer, autumn and winter are 0.72, 0.89, 0.88 and 0.93, respectively (Adem^18^).

Then using Eqs. (2) and (3), it is found that:

4 $$\Delta \varepsilon =-\frac{{\alpha }_{1}+2{\alpha }_{2}{T}^{*}}{{B}_{2}}\Delta {T}^{*}$$

In order to compute the first order increase, the authors selected a constant value of T^*^, i.e. the basic temperature of the cloud cover used in the TCM^23^: 263.5 K. Also, the standard temperature (at sea level) ${T}_{a0}=288 \; \text{K}$ and $\beta =6.5 \,\text{K} \, \text{km}^{-1}$ are used. $\Delta {T}^{*}$ is assumed as the increase of temperature at the mid-troposphere (${T}_{m}$), which is the main variable of the model. The result is a negative correlation with a single coefficient:

5 $$\Delta \varepsilon =-0.0126 \, {\text{K}}^{-1}\Delta {T}_{m}$$

**Figure 1:**
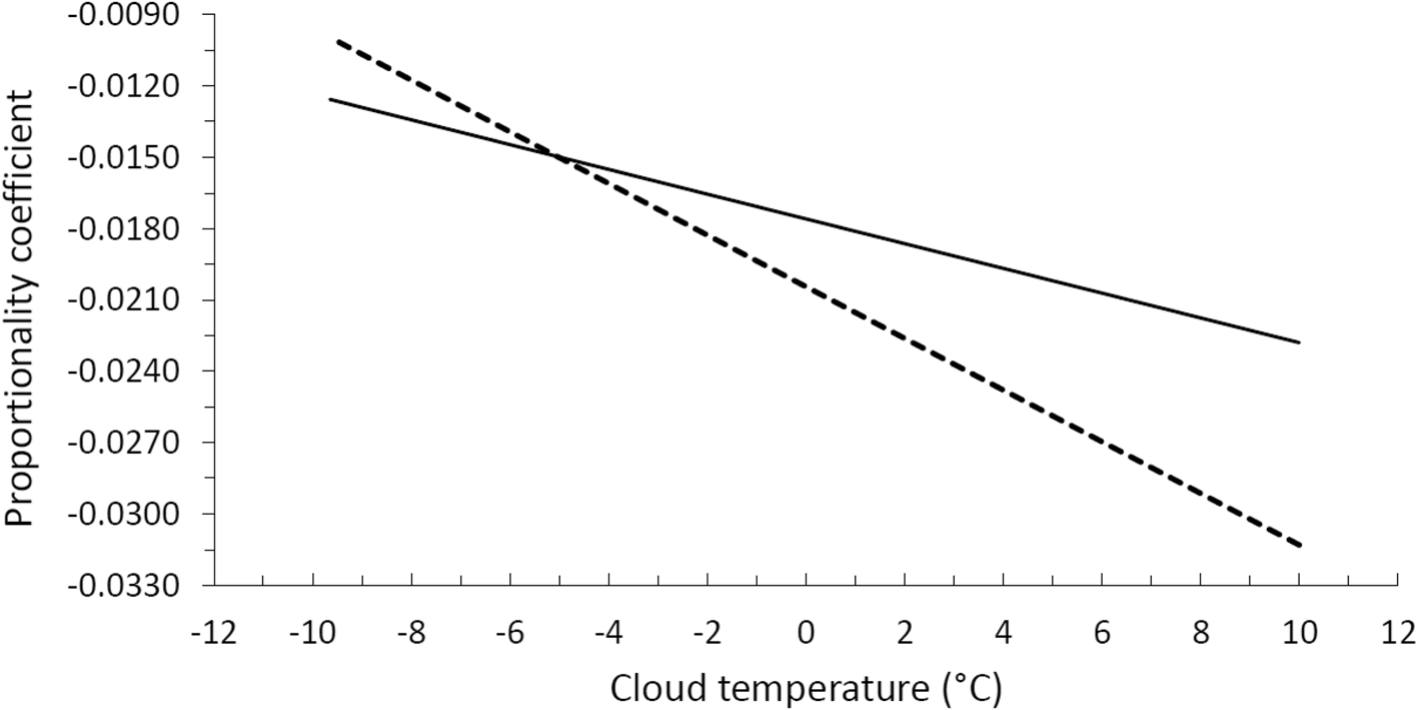
Proportionality coefficient computed from (4) as a function of the cloud temperature for two values of the tropospheric lapse rate $6.5\, \text{K} \, \text{km}^{-1}$ (continuous line) and $4.5\, \text{K} \, \text{km}^{-1}$ (dashed line).

This formula establishes that a 1 °C heating or cooling of the mid troposphere, decreases or increases respectively the total clouds cover by 1.26 percentage points (pp). The numerical value of proportionality coefficient in Eq. (5) depends on the reference values used; therefore, we have calculated this coefficient using other reference values for the cloud temperature and for $\beta$. Gultepe and Isaac^24^ have studies from the aircraft observations of cloud droplet number concentration in three types of cloud systems over Canada, namely Arctic clouds, maritime boundary-layer clouds, and winter storms as function of cloud temperature, their results show that the droplet number concentration reaches maximum values at about – 10 °C (~ 263 K), 2 °C (275 K) and 10 °C (283 K). We assume these temperatures as representative of dense clouds, which may be important in the climate. On the other hand, Mokhov and Akperov^25^ have estimated the tropospheric lapse rate and its relationship with surface temperature on a global scale using monthly mean data from the NCEP/NCAR reanalysis. They find that in tropical and middle latitudes $\beta$ is around $6.0 \, \text{K} \, \text{km}^{-1}$ over the ocean and between $5.5\, \text{K} \, \text{km}^{-1}$ and $6.5\, \text{K} \, \text{km}^{-1}$ over the continent; in polar latitudes its value ranges from $5.5\, \text{K} \, \text{km}^{-1}$ to $4.5\, \text{K} \, \text{km}^{-1}$ and even $3.5\, \text{K} \, \text{km}^{-1}$ in Antarctica. Taking into account these studies, we have calculated the proportionality coefficient from Eq. (4) as a function of the cloud temperature for two values of the tropospheric lapse rate; the result is shown in Fig. 1. For the case of a lapse rate equal to $6.5\, \text{K} \, \text{km}^{-1}$, the coefficient varies in the range of − 0.0126 to − 0.0220 K^−1^ (continuous line) and for the case of $4.5\, \text{K} \, \text{km}^{-1}$ (dashed line), between − 0.01 and − 0.0308 K^−1^; being in both cases always negative.

## Second parameterization

The precipitable water in the atmosphere can be expressed as

6 $$w={\int }_{0}^{H}{\rho }^{*}{q}^{*}dz$$

where *H* is the tropospheric height, ${\rho }^{*}$ is the air density and ${q}^{*}$ is the specific humidity, the asterisk means that the variable depends on z.

Using the state equation, the partial density of water vapor is:

7 $${\rho }^{*}{q}^{*}=\frac{0.622}{R{T}^{*}}{e}^{*}$$

where $R$ is the dry air gas constant and ${e}^{*}$ is the partial vapor pressure.

TCM uses a linear profile for ${T}^{*}$ in the troposphere with a lapse rate ($\beta$) constant; however, to facilitate the integration of Eq. (6), we used, as in Mendoza et al.^26^, an exponential profile for ${T}^{*}$ and ${e}^{*}$, in this way:

8 $${T}^{*}\left(z\right)={T}_{a}\mathrm{exp}\left(-\frac{\beta z}{{T}_{a0}}\right)$$

9 $${e}^{*}\left(z\right)={e}_{a}exp\left[-\left({k}_{w}+\beta /{T}_{a}\right)z\right]$$

with the surface values ${T}_{a0}={T}^{*}(z=0)$ and ${e}_{a}={e}^{*}(z=0)$, and

10 $${k}_{w}=5.8\times {10}^{3} \, \text{K}\left(\beta /{T}_{a0}^{2}\right)-\beta /{T}_{a0}+0.055 \, {\text{km}}^{-1}$$

The difference between the linear and the exponential profile for ${T}^{*}$ at 6 km altitude is only 1%, and 4.6% at the beginning of the tropopause (at 12 km altitude). Taking the standard values of ${T}_{a}$, we found ${k}_{w}=0.487\, \text{km}^{-1}$.

Substituting Eqs. (8) and (9) in (7) and the result in (6), the integral yields

11 $$w=\frac{0.622}{{k}_{w}R{T}_{a}}\left(1-{e}^{{-k}_{w}H}\right){e}_{a}$$

According to the definition of saturation vapor pressure (${e}_{s}$), ${e}_{a}$ can be expressed as:

12 $${e}_{a}={f}^{*}\left(z=0\right){e}_{s}\left({T}_{a}\right)$$

Adem^18^ found that:

13 $${f}^{*}\left(z=0\right)={f}_{m}+{A}_{0}$$

where ${A}_{0}$ is a parameter dependent on the season. In Eq. (13) we use the annual average for ${A}_{0}$, which is 21.58% and the standard deviation is 1.7%.

Substituting (13) in (12) and the resulting expression in Eq. (11), we obtain:

14 $$w=M\left({f}_{m}+{A}_{0}\right)\frac{{e}_{s}\left({T}_{a}\right)}{{T}_{a}}$$

where

15 $$M=\frac{0.622}{{k}_{w}R}\left(1-{e}^{-{k}_{w}H}\right)$$

Differentiating Eq. (14), assuming in Eqs. (8) and (9) to $\beta$ as constant, and using the Clausius–Clapeyron equation:

16 $$\frac{d{e}_{s}\left({T}_{a}\right)}{{e}_{s}\left({T}_{a}\right)}=\frac{{L}_{v}}{{R}_{v}{T}_{a}}\frac{d{T}_{a} }{{T}_{a}}$$

we have

17 $$\frac{dw}{w}=\left(\frac{{L}_{V}}{{R}_{V}{T}_{a}}-1\right)\frac{d{T}_{a}}{{T}_{a}}+\frac{d{f}_{m}}{{f}_{m}+{A}_{0}}$$

where ${L}_{v}$ is the latent heat of vaporization and ${R}_{v}$ the vapor gas constant.

Integrating Eq. (17) between a reference state ${w}_{0}, {T}_{a0}$ and ${f}_{m0}$ and the state $w={w}_{0}+\Delta w, {T}_{a}={T}_{a0}+\Delta {T}_{a}$ and ${f}_{m}={f}_{m0}+\Delta {f}_{m}$, where $\Delta {T}_{a}/{T}_{a0}, \Delta w/{w}_{0}$ and $\Delta {f}_{m}/{f}_{m0}$ are $\ll 1$, and considering that ${ln}\left(1+x\right)\approx x$ for $x\ll 1$, we obtain:

18 $$\Delta {f}_{m}=\frac{{T}_{a0}}{M{e}_{s}({T}_{a0})}\Delta w+\frac{{w}_{0}}{M{e}_{s}({T}_{a0})}\left[1-\frac{{L}_{v}}{{R}_{v}{T}_{a0}}\right]\Delta {T}_{a}$$

In Eq. (18), considering Eq. (14) evaluated at the reference state, we have used the equality: ${f}_{m0}+{A}_{0}={w}_{0}{T}_{a0}/(M{e}_{s}\left({T}_{a0}\right))$. Using then Eq. (3) in (18), we found:

19 $$\Delta \varepsilon =\frac{{T}_{a0}}{{B}_{2}M{e}_{s}({T}_{a0})}\Delta w+\frac{{w}_{0}}{{B}_{2}M{e}_{s}({T}_{a0})}\left[1-\frac{{L}_{v}}{{R}_{v}{T}_{a0}}\right]\Delta {T}_{a}$$

Mendoza et al.^20^ found for the NH that in annual and hemispheric average, ${f}^{*}$ decreases as the temperature increases in the troposphere; for example, at 850 mb, where ${f}^{*}$ is ~ 63.9%, an increase of 1 °C is associated with 3 pp decrease in ${f}^{*}$, while on the surface, where ${f}^{*}$ is ~ 78.3%, the decrease is only 0.73 pp. Using this condition, Mendoza et al.^20^ determine that the precipitable water in the troposphere can be expressed as:

20 $$w={w}_{0}{e}^{0.0282\, {\text{K}}^{-1}\Delta {T}_{a}}$$

where ${w}_{0}=1.825 \, \text{cm}$ is the typical (annual and global, long-term average) of $w$. The value of $0.0282 \, {\text{K}}^{-1}$ in the exponent of Eq. (20) is related to the fact that the ${f}^{*}$ is not conserved; that is: ${f}^{*}$ decreases (increases) as the air temperature increases (decreases). For the case in which ${f}^{*}$ is conserved (at every level) with a change of temperature, the exponent in Eq. (20) is higher and its value is $0.0557 \, {\text{K}}^{-1}$ (Mendoza et al.^20^).

Therefore, from Eq. (20)

21 $$\Delta w=w-{w}_{0}={w}_{0}\left({e}^{0.0282\, {\text{K}}^{-1}\Delta {T}_{a}}-1\right)$$

Usually, $$0.0282 \, {\text{K}}^{-1}\Delta {T}_{a}\ll 1$$ so:

22 $${e}^{0.0282\, {\text{K}}^{-1}\Delta {T}_{a}}\approx 1+0.0282\, {\text{K}}^{-1}\Delta {T}_{a}$$

And thus

23 $$\Delta w=0.0282\, {\text{K}}^{-1}{w}_{0}\Delta {T}_{a}$$

According to Eq. (23), which is an approximation of Eq. (20); a 1 K increase in ${T}_{a}$ is associated with an increase in precipitable water of 0.05 cm when ${f}^{*}$ is not conserved. When ${f}^{*}$ is conserved, we must change the coefficient of $0.0282\, {\text{K}}^{-1}$ in Eq. (23) by $0.0557\, {\text{K}}^{-1}$, in this case an increase of 1 K in ${T}_{a}$ is associated with an increase in precipitable water of 0.10 cm; that is, more water vapor must be supplied to the atmospheric column to keep the relative humidity constant. As we will see later, a higher moisture content in the atmosphere, keeping the relative humidity constant, will be associated with less decrease in cloud cover as the temperature increases compared to the case where the relative humidity is not conserved, where the decrease in cloud cover is greater.

Substituting Eq. (23) in (19), we have

24 $$\Delta \varepsilon =\frac{0.0282\, {\text{K}}^{-1}{T}_{a0}+1-\frac{{L}_{v}}{{R}_{v}{T}_{a0}}}{{B}_{2}M{e}_{s}({T}_{a0})}{w}_{0}\Delta {T}_{a}$$

Using in Eq. (24) the constants and the standard or typical values given above, in MKS (and *K*) units, and the following parameter values: ${T}_{{a}_{0}}\equiv 288 \, \text{K}$, ${k}_{w}=4.87\times {10}^{-4}{ \text{m}}^{-1}$, $H=1.1\times {10}^{4}\, \text{m}$, ${L}_{v}=2.257\times {10}^{6} \, {\text{J}}\, \text{kg}^{-1}$, $R=287.06 \, {\text{J}}\, \text{kg}^{-1} \, {\text{K}}^{-1}$, ${R}_{V}=461.5 \, {\text{J}}\, \text{kg}^{-1} \, {\text{ K}}^{-1}$, ${B}_{2}=0.5$, ${e}_{s}=17 \; \text{mb}=1.7\times {10}^{3} \, \text{kg} \, {\text{m}}^{-1} \, {\text{s}}^{-2}$, $M=4.428\, {\text{m}}^{-1}\, {\text{s}}^{2} \, \text{K}$, ${w}_{0}=18.25 \, {\text{kg}} \, \text{m}^{-2}$.

we obtain for the case in which the relative humidity is not conserved:

25 $$\Delta \varepsilon =-0.0381\, {\text{K}}^{-1}\Delta {T}_{a}$$

In the case where the relative humidity is conserved, the factor $0.0282\, {\text{K}}^{-1}$ in Eq. (24) must be changed to $0.0557\, {\text{K}}^{-1}$, resulting in:

26 $$\Delta \varepsilon =-0.0075\Delta {T}_{a}$$

This way, when ${f}^{*}$ is not conserved (Eq. 25), an increase of 1 K in ${T}_{a}$ is associated with a decrease of 3.81 pp in the cloud cover and when ${f}^{*}$ is conserved (Eq. 26), this decrease is less: 0.75 pp.

Providing that ${T}^{*}$ at $z=H$ remains unchanged, like a pivot of the temperature profile^20^, we have

27 $$\Delta {T}_{a}=2\Delta {T}_{m}$$

Therefore, Eq. (25) can be expressed as

28 $$\Delta \varepsilon =-0.076\, {\text{K}}^{-1}\Delta {T}_{m}$$

And Eq. (26) as

29 $$\Delta \varepsilon =-0.015\, {\text{K}}^{-1}\Delta {T}_{m}$$

## Results and discussion

Equation (19) indicates that the increase of $\varepsilon$ is equal to a linear combination of the increases of precipitable water and of air surface temperature. In this way, if these increases can be predicted, Eq. (19) calculates the increase of $\varepsilon$. If only the mean tropospheric temperature increase can be predicted, Eq. (24) calculates the cloudiness increase. The negative sign in Eqs. (25) and (26), which are derived from Eq. (24), comes from the Clausius–Clapeyron equation because the term $\frac{{L}_{v}}{{R}_{v}{T}_{a0}}$ is greater than $0.0282\, {\text{K}}^{-1}{T}_{a0}+1$ for the case in which the relative humidity is not conserved, and that $0.0557\, {\text{K}}^{-1}{T}_{a0}+1$ for the case in which the relative humidity is conserved.

To verify our parameterizations given by Eqs. (25) and (26), we will correlate the cloudiness data with the surface air temperature data. For this we will consider three types of clouds, where each type of cloud is formed in a layer at a different level: low clouds ${\varepsilon }_{L}$, medium clouds ${\varepsilon }_{M}$ and high clouds ${\varepsilon }_{H}$. According to Tompkins and Di Giuseppe^27^, in the calculation of the total clouds cover $\varepsilon$ there are three options in their formation: clouds that were formed through coherent dynamic processes (mainly convection) and that overlap in a maximum way, in this case the total cloud cover is $\varepsilon =Max\left\{{\varepsilon }_{L}, {\varepsilon }_{M}, {\varepsilon }_{H}\right\}$; clouds that are formed in a random way, in a way this represents that the processes that take place in their formation at each level are not well known, in this case averaging a very large number of possible overlap scenarios, the total cloud cover will be given on average by $\varepsilon =1-\left(1-{\varepsilon }_{L}\right)\left(1-{\varepsilon }_{M}\right)(1-{\varepsilon }_{H})$; finally, the third option considers that the clouds in each layer overlap minimally, in theory this could occur if the presence of a cloud at one level leads to a lower probability that there is a nearby cloud at the other level, possibly due to processes of suppression, in this case we have $\varepsilon =Min\left\{{\varepsilon }_{L}+{\varepsilon }_{M}+{\varepsilon }_{H},1\right\}$. Considering the database of the International Satellite Cloud Climatology Project (ISCCP: https://isccp.giss.nasa.gov/), we can take the following typical fractional values for cloud cover at each of the three levels: ${\varepsilon }_{L}=0.273$, ${\varepsilon }_{M}=0.206$ and ${\varepsilon }_{H}=0.132$; thus in the first case $\varepsilon =Max\left\{{\varepsilon }_{L}, {\varepsilon }_{M}, {\varepsilon }_{H}\right\}=0.273$, in the second $\varepsilon =1-\left(1-{\varepsilon }_{L}\right)\left(1-{\varepsilon }_{M}\right)\left(1-{\varepsilon }_{H}\right)=0.499$ and in the third $\varepsilon =Min\left\{{\varepsilon }_{L}+{\varepsilon }_{M}+{\varepsilon }_{H},1\right\}=0.611$. According to this result, the first option minimizes cloud cover, resulting in low cloud cover as is. The second option may underestimate the total cloud cover by assuming that each cloud at one level overlaps with a cloud at another level; however, it may be a good option if the physical processes that take place in the formation of clouds at each level are unknown. The third option can lead to the error of overestimating the cloud cover; but it may be an acceptable option when considering the total cloud cover formed only by low and medium clouds, since the increase in low clouds suppresses the increase in medium clouds due to the lack of vapor at this level, which condensed at the level of low clouds, the opposite occurs when there is a decrease in low clouds, in which case the excess vapor at this level can condense at the level of the middle clouds, giving an increase in clouds at this level^20^. In Mendoza et al.^20^ we call the cloud cover formed by low and medium clouds, relevant cloud cover ${\varepsilon }_{R}$ (because it is the most important component of the planetary albedo), in that work ${\varepsilon }_{R}$ was calculated using random superposition.

**Figure 2:**
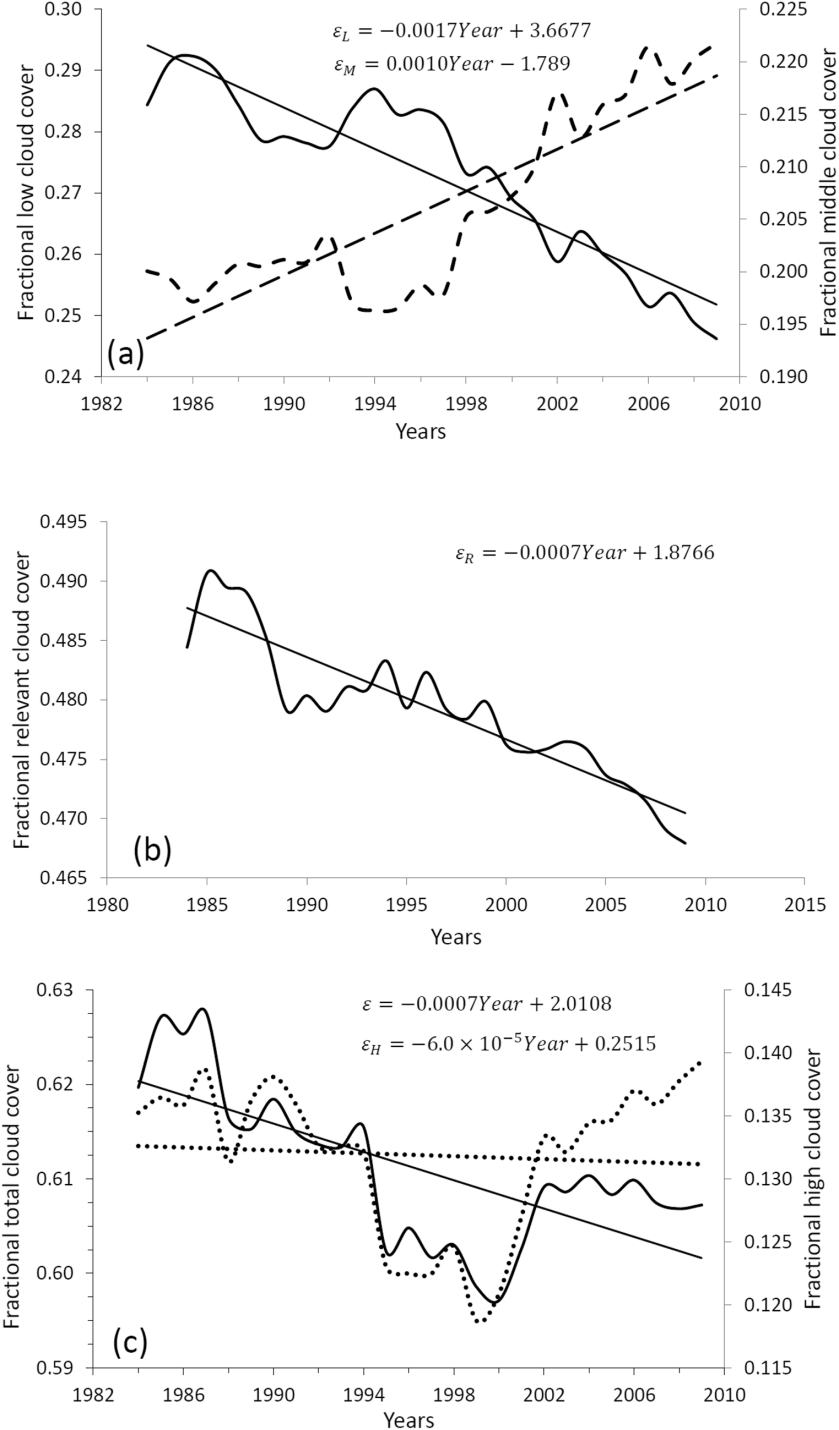
Global and annual average of fractional cloud cover during the 26-year period from 1984 to 2009 obtained from the ISCCP database. Part (**a**) corresponds to ${\varepsilon }_{L}$ (solid curve) and ${\varepsilon }_{M}$ (dashed curve); the part (**b**) to ${\varepsilon }_{R}=Min\left\{{\varepsilon }_{L}+{\varepsilon }_{M},1\right\}$ and the part (**c**) to $\varepsilon =Min\left\{{\varepsilon }_{L}+{\varepsilon }_{M}+{\varepsilon }_{H},1\right\}$ (continuous curve) and to ${\varepsilon }_{H}$ (dotted curve).

Figure 2 shows the evolution of the global and annual average fractional cloud cover over the 26-year period from 1984 to 2009 obtained from the ISCCP database. Part (a) corresponds to the ${\varepsilon }_{L}$ (continuous curve) and ${\varepsilon }_{M}$ (dashed curve); part (b) to ${\varepsilon }_{R}$ calculated as ${\varepsilon }_{R}=Min\left\{{\varepsilon }_{L}+{\varepsilon }_{M},1\right\}$ and part (c) to $\varepsilon =Min\left\{{\varepsilon }_{L}+{\varepsilon }_{M}+{\varepsilon }_{H},1\right\}$ (continuous curve) and ${\varepsilon }_{H}$ (dotted curve). Part (a) shows a clear anti-correlation between low and middle clouds due to the process of suppression (or the opposite process called instauration) of vapour at the middle cloud level, because of this process we have used case three described above in the calculation of ${\varepsilon }_{R}$ and $\varepsilon$. It is important to note that unlike the low and middle clouds, the high clouds, in part (c), do not show any long-term trend (dotted straight line). The linear trend of ${\varepsilon }_{L}$ (continuous straight line) is − 0.17% per year, while that of ${\varepsilon }_{M}$ (dashed straight line) is 0.10% per year, thus the trend of ${\varepsilon }_{R}$ turns out to be − 0.07% per year, lower than that of ${\varepsilon }_{L}$. On the other hand, the long-term linear trend of the total cloud cover (continuous straight line) is − 0.07% per year, the same as that of ${\varepsilon }_{R}$.

**Figure 3:**
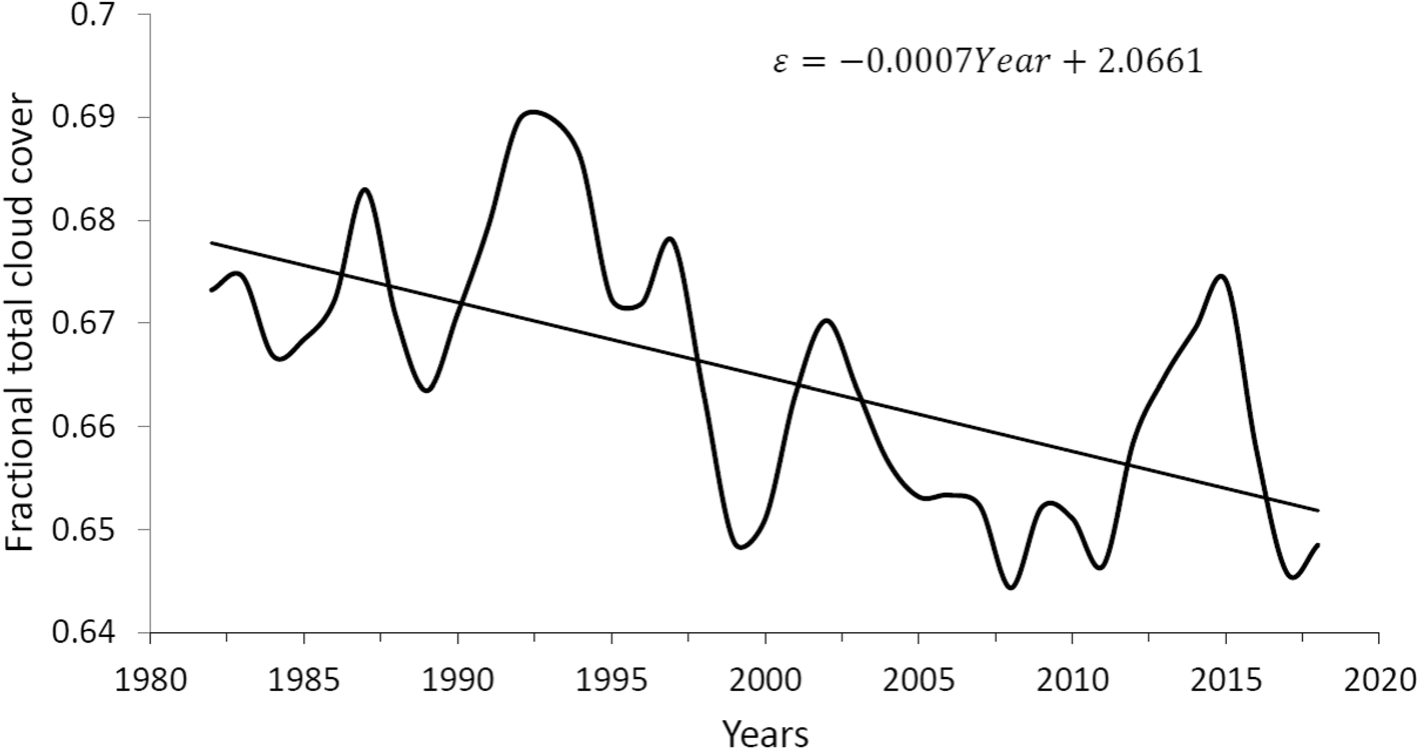
Global and annual average of total cloud cover during the 37-year period from 1982 to 2018, obtained from the CLARA-A2.1 monthly global database with a 25-month running average, thus reducing its deviation standard 2.97 to 1.28 pp.

**Figure 4:**
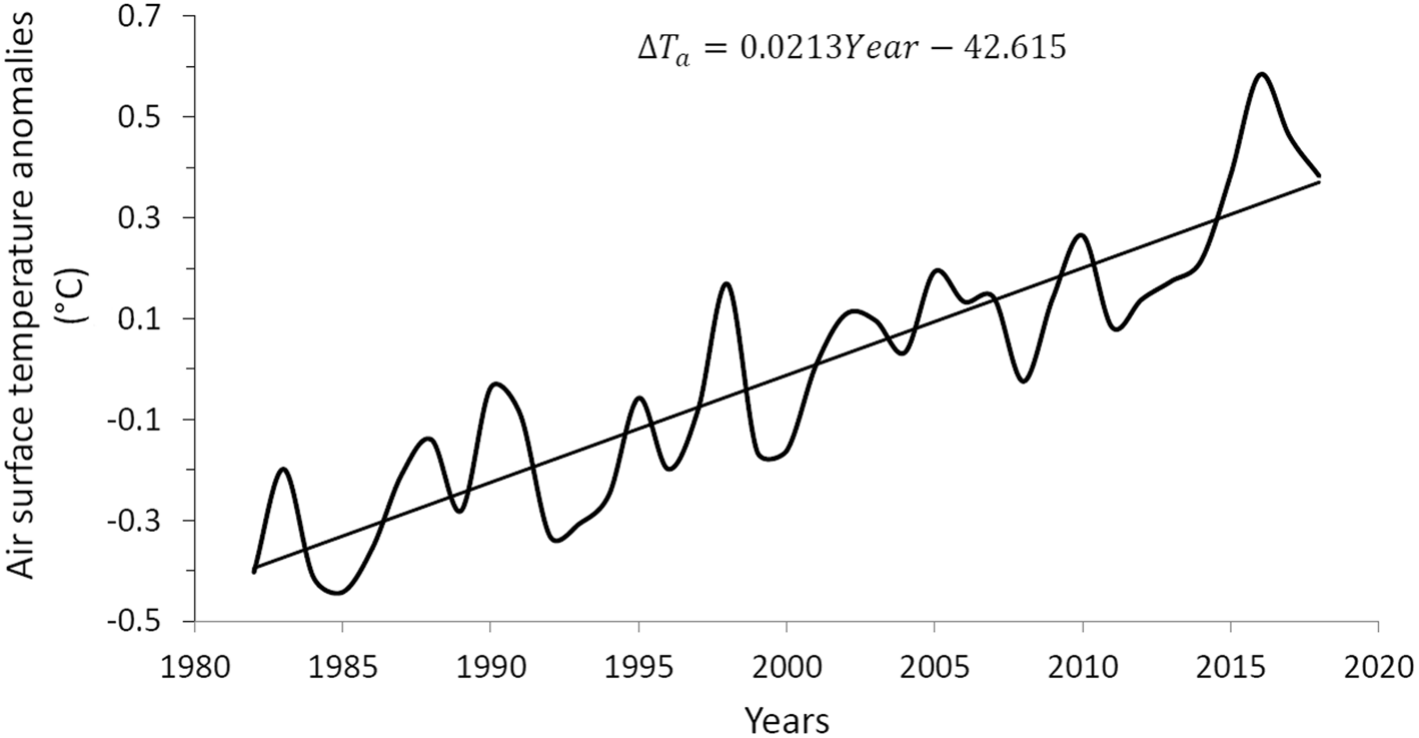
Global and annual anomaly (which is equivalent to what in this work we have called increase $\Delta$) of the air temperature at 2 m above the surface, obtained from the ERA5 Reanalysis for the period of 37 years from 1982 to 2018.

A more extensive and recent period of total cloud cover is constituted by the global monthly database of 37 years from 1982 to 2018 reported by CLARA-A2.1^28^, which shows an average of 66.5% with a standard deviation of 2.97 pp, which represents a greater monthly variability than the ISCCP database, which has a standard deviation of 0.9 pp. In this way, in order to have some consistency in the use of the two databases, we have modified the CLARA-A2.1 database by making a 25-month running average, thus reducing its standard deviation to 1.28 pp. Figure 3 shows the annual average of the total cloud cover of this filtered database, in this case the long-term linear trend is also − 0.07% per year as in Fig. 2, part (c). For its part, the global and annual anomaly (which is equivalent to what in this work we have called increase) of the air temperature at 2 m above the surface is taken from the ERA5 Reanalysis (https://www.ecmwf.int/en/forecasts/datasets/reanalysis-datasets/era5) for the period 1982 to 2018 and is shown in Fig. 4.

**Figure 5:**
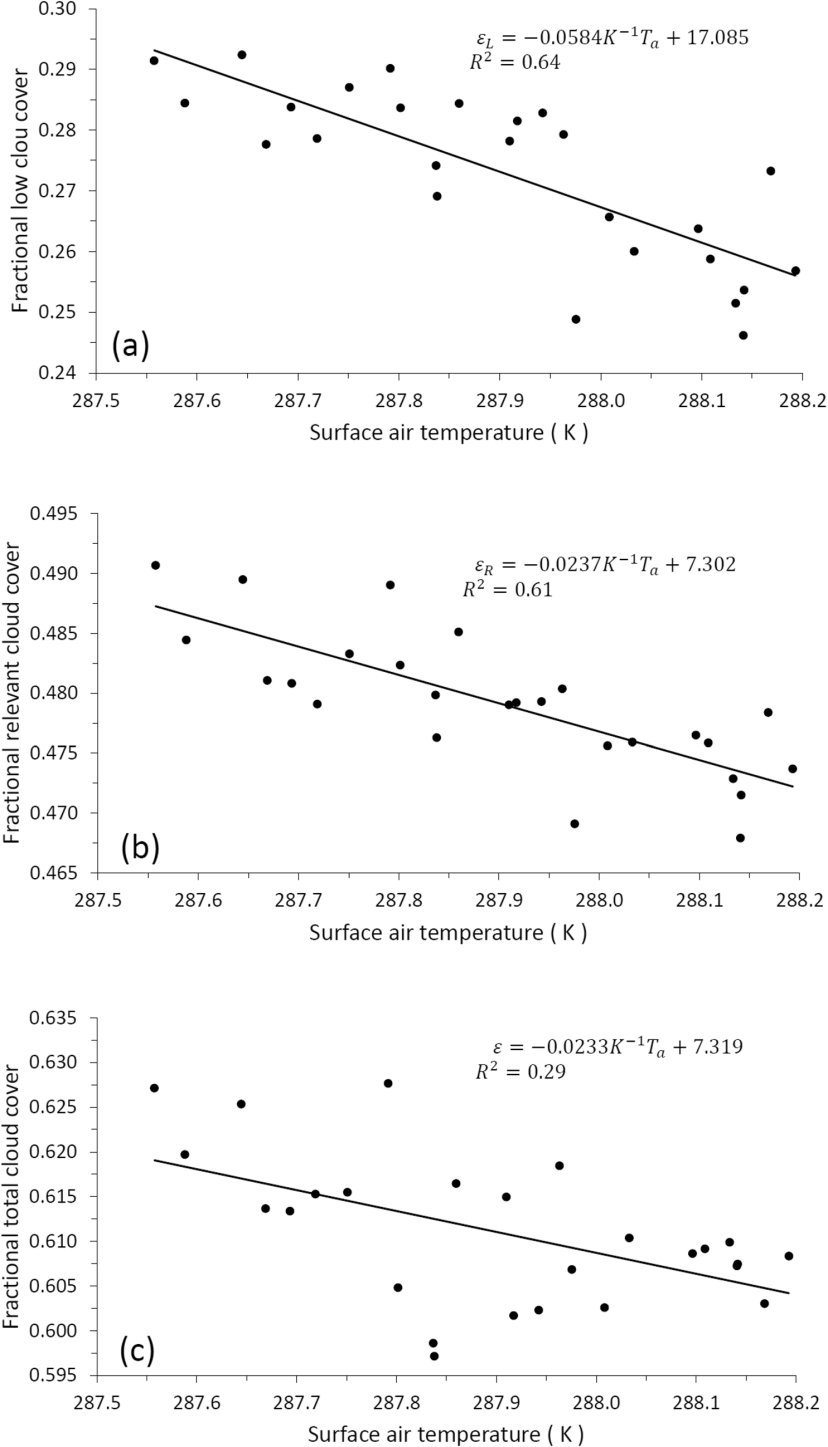
Correlation between cloud cover (from ISCCP) and absolute surface air temperature, obtained by adding to the temperature anomaly in Fig. 4, the standard temperature ${T}_{{a}_{0}}=288 \, \text{K}$. The straight line shows the long-term trend, whose equation and correlation $({R}^{2})$ are shown in the upper right. Part (**a**) corresponds to low cloud cover, (**b**) to relevant cloud cover and (**c**) to total cloud cover.

**Figure 6:**
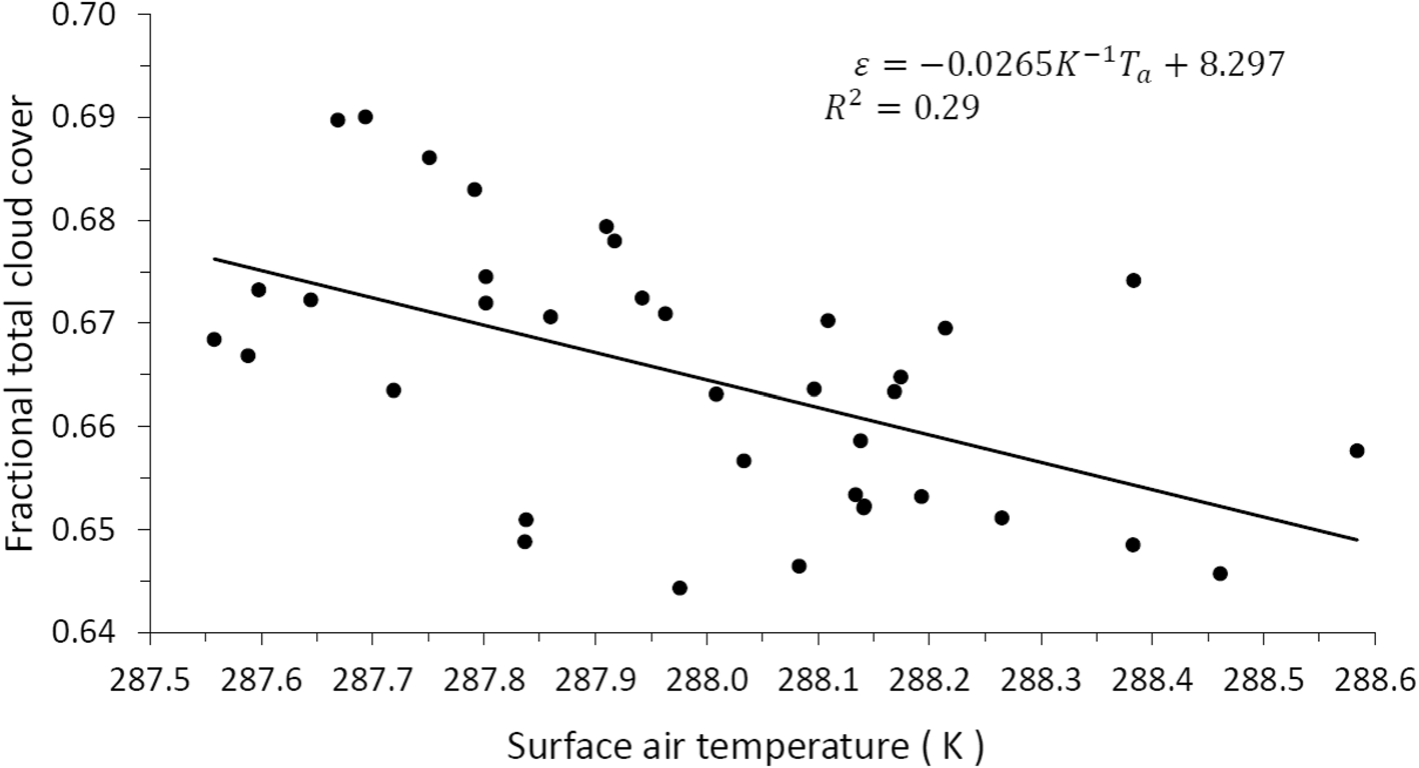
Correlation between the total cloud cover of the CLARA-A2.1 database shown in Fig. 3 and the absolute surface air temperature; in this case the finite increment of $\varepsilon$ is $\varepsilon =-0.0265\, {\text{K}}^{-1}\Delta {T}_{a}$, with the same linear correlation (${R}^{2}=0.29$) as that shown in part (c) of the Fig. 5.

Figure 5 shows the correlation between cloud cover (from ISCCP) and absolute surface air temperature, obtained by adding to the temperature anomaly in Fig. 4, the standard temperature ${T}_{{a}_{0}}=288 \, \text{K}$; the line shown is the long-term trend line, whose equation and correlation $({R}^{2})$ are shown in the upper right. According to part (a) of Fig. 5, a finite increase in ${\varepsilon }_{L}$ along the long-term trend line is given by $\Delta {\varepsilon }_{L}=-0.0584\, {\text{K}}^{-1}\Delta {T}_{a}$, the value of this negative slope is higher than the case in which the relative humidity is not conserved (Eq. 25) and in which it is conserved (Eq. 26); part (b) shows that a finite increase in the relevant clouds given by $\Delta {\varepsilon }_{R}=-0.0237\, {\text{K}}^{-1}\Delta {T}_{a}$ has a negative slope whose value is between the case in which the relative humidity does not is conserved (Eq. 25) and in which it is conserved (Eq. 26). The slope in $\Delta {\varepsilon }_{R}$ results in an absolute value less than the slope of $\Delta {\varepsilon }_{L}$, because an increase in the middle clouds gives a positive slope (part not shown here), that is, $\Delta {\varepsilon }_{M}=0.0347\, {\text{K}}^{-1}\Delta {T}_{a}$. Part (c) of Fig. 5 shows that in an increment of $\varepsilon$, given by $\Delta \varepsilon =-0.0233\, {\text{K}}^{-1}\Delta {T}_{a}$, the negative slope has a value very close to that of the slope of the increase in relevant clouds, but with a significantly lower linear correlation $({R}^{2}=0.29)$ than that which occurs in the cases of low clouds $({R}^{2}=0.64)$ and relevant clouds $({R}^{2}=0.61)$, which is due to the noise introduced by the high clouds, since they show a complete dispersion without any correlation with the temperature. Figure 6 shows the correlation between the total cloud cover of the CLARA-A2.1 database shown in Fig. 3 and the absolute surface air temperature shown in Fig. 4; in this case the increment of $\varepsilon$ is $\Delta \varepsilon =-0.0265\, {\text{K}}^{-1}\Delta {T}_{a}$, with the same linear correlation $({R}^{2}=0.29)$ as that shown in part (c) of the Fig. 5.

Brient and Bony^29^, analyze the positive feedback of the low clouds that are generated with a low resolution version of the IPSL-CM5A climate model used in CMIP5 when a simulation is carried out in which the CO_2_ concentration increases by 1% each year. This feedback is associated with an increase in temperature, a decrease in low clouds and an increase in the net radiative cloud forcing (NRCF) at the top of the atmosphere; On a global scale, the decrease in low clouds results from $0.90\% \, {\text{K}}^{-1}$ and the increase in the NRFC of $0.5 \, {\text{Wm}}^{-2} \, {\text{K}}^{-1}$ dominated by the short-wave radiation component ($+1.3 \, {\text{Wm}}^{-2} \, {\text{K}}^{-1}$). In our parameterizations, the decrease in total cloud cover associated with an increase in temperature ($\Delta \varepsilon /\Delta {T}_{a}$) is $3.80\% \, \text{K}^{-1}$ when relative humidity is not conserved (Eq. 25) and $0.75\% \, \text{K}^{-1}$ when relative humidity is conserved (Eq. 26), this last result is very close to that reported by Brient and Bony^29^. According to Qu et al.^30^, climate models are driven by large-scale changes, tropical inversion, increment in latent heat flux, and the increase of a warming vertical heat. They are represented by the temperature at the surface and are related with the decrease of low-level clouds. This allows more solar radiation heating the surface, which agree with our results. A decrease in low level clouds is related to the sensitivity of the models to temperature increase^31–34^.

By comparing Eq. (5) with (29) we notice that arrive through different ways to the negative linear correlation between changes of cloudiness and tropospheric temperature, with proportionality constants that are very similar, for the case where relative humidity is conserved.

The cloud cover increase can generate surface warming by longwave greenhouse, or cooling by shortwave reflection due to the cloud high albedo. When the reflection effect is dominant, which occurs on a global scale, Eqs. (5), (28) or (29) lead to a predominant positive feedback^13,14^. If in a climate change the temperature increases, the cloud cover decreases, more solar radiation reaches the surface, and the temperature increases even more. In regions where the greenhouse effect dominates (for instance, in polar regions), these formulas indicate that, given an increase in temperature, the cloud cover reduction allows an increase of outgoing longwave radiation, which propitiates a damping of the temperature increase, that is, we are in the case of a negative feedback.

## Data Availability

The datasets generated during and/or analyzed during the current study are available from the corresponding author on reasonable request.
